# Dujardin-Beaumetz on Hypnotics

**Published:** 1886-03

**Authors:** 


### Dujardin-Beaumetz on Hypnotics.

In a series of lectures, entitled, "Conference de Therapeutiques," Dujardin-Beaumetz has taken up various therapeutical subjects. In a recent lecture, which appears in the *Bulletin Général de Thérapeutique*, for Oct. 30, 1885, he discourses on the "new hypnotics." We transcribe some of his observations:

Chloral appears to act on the heart, and, as has been affirmed by Gubler, it is a heart poison in large doses. In all febrile diseases of a congestive form, chloral is far superior to opium for the production of sleep; as in typhoid pneumonia, and alcoholic delirium. On the contrary, this remedy is contra-indicated in cardiac affections, especially in troubles of the aortic orifice; here opium is much better. Chloral is a most useful remedy in certain forms of intoxication, especially in poisoning by strychnine, in delirium tremens, and uræmic convulsions, but in these it is still inferior to paraldehyde.

The sleep produced by paraldehyde is analogous to that produced by chloral. It is usually calm, but in some instances the sleep is preceded by excitement. The assertion of Quinquad and Henocque that it acts on the hæmoglobin, producing methemoglobin, has been disproved by Hayem, who has shown that it does not affect the coloring matter of the blood. It is eliminated almost entirely by the lungs, but when large doses are given some portion escapes in the sweat.

One of the most interesting facts regarding the action of paraldehyde is its antagonism to strychnine, which was first demonstrated by the Italian physicians, and since confirmed by

Coudray. Dr. Dujardin-Beaumetz relates some experiments of his own, proving this antagonism. Ether, chloral, chloroform, have similar powers in this respect. They all act, as does paraldehyde, on the cells of the nervous matter. We know that strychnine, also, stimulates the cells of the cerebro-spinal axis. It consequently happens that when the nerve elements are acted on by one agent, they will not receive an impression from another, and thus, in a strictly physiological and scientific manner, can be explained the antagonisms of these several remedies and strychnine.

Compared with chloral, paraldehyde has these advantages:

It is less irritant, and better supported by the stomach. It is not a heart poison. It is a more efficient antidote to strychnine. But it has less analgesic action, and less power to relieve pain than chloral, and hence when insomnia is caused by pain the latter is preferable, and morphine is still more efficient. In nervous insomnia, in that due to the abuse of alcoholic drinks, paraldehyde is much superior to chloral. Especially is paraldehyde most useful in the different forms of mental disorder, as Drs. Keraval and Nerkam have shown by numerous trials. They have also shown that it is a valuable hypnotic in certain cases of insomnia with the excitement occurring in the course of some cerebral affections, in the convulsive neuroses, and especially in epileptic crises, and in the multiform manifestations of hysteria. Dr. Dujardin-Beaumetz has also treated many cases of morphiomania by paraldehyde, giving three or four grammes a day (forty-five to sixty minims). It does not appear to lose its effect by repetition, since the same results have been obtained through months of treatment.

Mr. G. F. Hodgson, an English surgeon, has had experiences with paraldehyde which supplement the observations of Dujardin-Beaumetz (*British Medical Journal*, July, 1885). His report is based on the experience gained from the use of about two quarts of the agent in a variety of cases. Mr. Hodgson finds it to be a hypnotic of great value, in that it produces sleep like the natural state, promptly, and without any unpleasant after-effects. He regards it as the most appropriate hypnotic in the insomnia of gout, in mania, hypochondriasis, delirium tremens, migraine, and in the wakefulness of ordinary diseases. His prescription is as follows:

| | | |
|---|---|---|
| ℞ | Paraldehyde, - - - - | ʒ j. |
| | Spiritus chloroformi, - - - | ♏ xv. |
| | Pulv. tragacanth. comp., - - - | ℈ j. |
| | Syrup aurantii, - - - - | ʒ iv. |
| | Aquam, ad., - - - - | ℥ iij. |

M.

This dose is sufficient in the milder cases, but must be repeated in the more severe.—*Amer. Jour. Med. Sciences*, Jan., 1886.

